# War Questions—Payments for Hospital Patients

**Published:** 1917-10-27

**Authors:** 


### WAR QUESTIONS—PAYMENTS FOR HOSPITAL PATIENTS.

If there is one question upon which the nation as a whole has a clear view and wish, it is that under no circumstances shall sailors and soldiers, suffering from wounds or illness, be admitted to a hospital or other medical institution except as the guests, and at the cost of the State. Yet the Government has not faced this question boldly and completely, and the Conference called by the British Hospitals Association, reported in another column, will bring things to a head, and should finally place the matter on an adequate and permanent basis. All who have had to do with our fighting men everywhere, either in the fighting line, or when they have been stricken down and grievously injured, agree that they are splendid fellows, full of courage, cheerfulness, and manly self-restraint, and that the nation and people at home can never do too much for our warriors of all ranks. The present circumstances under which sailors and soldiers are admitted to our voluntary hospitals are fully dealt with in the discussions at the Conference, which will be found on pages 79-83. The meeting was large and informative, many of the leading London and provincial hospitals, the War Office, and the Ministry of Pensions being represented. It will be seen that the resolutions adopted supported (*a*) measures to obtain an immediate increase in the rates of payment for the treatment of wounded soldiers in civilian hospitals, and (*b*) that the scale of fees for patients sent by the Minister of Pensions should be such as would cover the whole cost of the treatment given, including payment for professional services. These resolutions mark an important departure on sound lines, and we have little doubt that they will receive generous consideration on a business basis from the War Office and the Government. It may be well to add that these resolutions, if properly enforced, will in no way affect the ordinary work of our voluntary hospitals, though they should tend to simplify and prepare the way for a reorganisation and amendments in the old methods of granting hospital relief in this country, which must be modified and greatly improved after the war.

The British Hospitals Association, in pursuance of the policy for which it was originally founded in 1884, did good service in calling the Conference at Westminster Hospital on the 19th inst. The representative nature of the attendance showed that the subjects dealt with had great interest for hospital managers up and down the country, and we look forward to many similar meetings for the consideration of subjects arising out of the war which are likely to be numerous and far-reaching. The Rev. G. B. Cronshaw, Treasurer of the Radcliffe Infirmary, Oxford, who has done much useful work in the way of propaganda and development, took a leading part in the proceedings, and proved once more how useful is the work he has done, and is doing, for voluntary hospitals. There is a general opinion, we find, that the usefulness of the Association and its authority would be increased if a few of the really active leaders of hospital opinion on the boards of some of our principal Metropolitan hospitals were to be elected as members of the Council. Without being invidious as to the type of men indicated, we may mention the Hon. Sir Arthur Stanley, G.B.E., the new Treasurer of St. Thomas's Hospital, and Chairman of the Joint-Committee of the British Red Cross and Order of St. John. The Americans have an excellent rule which, we believe, applies to the British Hospitals Association, that the principal officers shall be elected annually, and, in view of the great interest now taken in all hospital affairs, this rule should be strictly followed, for it tends to keep the organisation alive and alert, and has many other advantages, as experience has proved both in the United States and in this country too. We heartily congratulate Mr. H. Wade Deacon, the present Chairman of Council, on the success of Friday's meeting, which reflects credit upon all associated with it, and is calculated to do real service.

